# Low-dose combination of ultramicronized palmitoylethanolamide and docosahexaenoic acid on neurosteroid and neuroinflammatory dysregulation in autism spectrum disorders

**DOI:** 10.1016/j.neurot.2025.e00816

**Published:** 2025-12-13

**Authors:** Fabiana Filogamo, Fabrizio Maria Liguori, Giovanna La Rana, Roberto Russo, Claudia Cristiano

**Affiliations:** Department of Pharmacy, University of Naples “Federico II”, via D. Montesano, 49, 80131, Naples, Italy

**Keywords:** Autism spectrum disorder, Docosahexaenoic acid, Palmitoylethanolamide, Allopregnanolone, Neuroinflammation

## Abstract

Several studies show that neurosteroids currently play a significant role in autism spectrum disorders (ASD). However, the pathway of neurosteroid synthesis involved in ASD remains unclear. This study aimed to investigate the crosstalk between autism and neurosteroids, focusing on the mechanism of allopregnanolone production. We used the BTBR T+ tf/J (BTBR) mouse, a well-established animal model of ASD that exhibits typical autism-like behaviors along with neuroinflammation. In the hippocampus of BTBR mice, we observed a marked overexpression of pregnenolone and a related reduction in allopregnanolone levels. This neurosteroid imbalance also appears to be associated with an inflammatory pattern and the manifestation of repetitive and asocial behaviors. The combination of low doses of ultramicronized palmitoylethanolamide (PEA-um) and docosahexaenoic acid (DHA) restores allopregnanolone production modulating neurosteroidogenesis. In association with neurosteroid modulation, this restoration reduces repetitive behaviors and improves social interactions in BTBR mice, also modulating the inflammatory profile with a significant reduction in proinflammatory cytokines and brain-derived neurotrophic factor (BDNF) levels in the hippocampus. These effects demonstrate an important role of the peroxisome proliferator-activated receptor alpha (PPAR-α), whose expression is particularly reduced in BTBR mice. In addition, the pivotal involvement of PPAR-α was further supported by administering a specific antagonist that abolished the advantageous effects of PEA-um ​+ ​DHA. Overall, our findings demonstrate the potential synergistic effect of the low-dose combination of PEA-um and DHA, confirming their therapeutic effect in ASD and the involvement of neurosteroids in their mechanism of action.

## Graphical abstract

**Figure:**
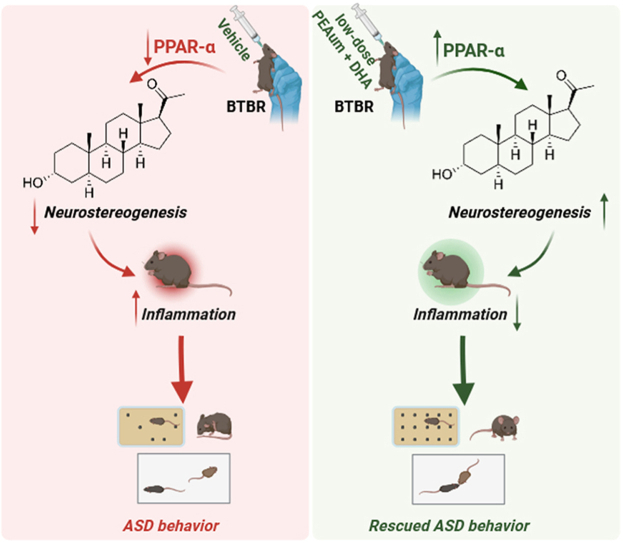

## Introduction

Neurosteroids are steroids that are synthesized in different areas of the central nervous system (CNS), such as the hippocampus and neocortex [1] and have direct and immediate effects on neuronal excitability, acting as endogenous modulators of the gamma-aminobutyric acid type A (GABA-A) receptor [2]. Neurosteroids are attracting considerable research interest in the field of neuropsychiatric disorders due to growing evidence of their therapeutic properties in various diseases [3]. Among the various neurosteroids present in the brain, allopregnanolone (ALLO, 5α-pregnan-3α-ol-20-one) is one of the most studied. ALLO is synthesized from progesterone through the enzymatic action of type I 5α-reductase (SRD5A1) and 3α-hydroxysteroid dehydrogenase (Akr1c4). Preclinical evidence has reported that alterations in ALLO levels plays a key role in several conditions such as neuropathic pain [4], depression [5,6], epilepsy [7] as well as its involvement in neurodegenerative diseases like Alzheimer's disease and multiple sclerosis [[8], [9], [10], [11], [12]]. The positive role of neurosteroids in autism spectrum disorder (ASD) has been also explored, but the mechanism by which this imbalance affects expression in ASD needs to be clarified.

ASD is a heterogeneous condition characterized by cognitive impairments, limited social interaction, and repetitive behaviour [13]. Several factors seem to contribute to the genesis of ASD, from a genetic predisposition to environmental factors; among these, one of the most widely approved hypotheses is the imbalance between the excitatory and inhibitory signaling in the brain, which leads to dysfunction of the GABAergic system.

However, the exact mechanisms by which these dysfunctions contribute to the pathophysiology of the autism remain unclear [14]. In particular, studies in the hippocampus of BTBR mice, a well-established animal model for ASD, have shown a reduced frequency of spontaneous inhibitory synaptic currents mediated by the GABA-A receptor, a key player in behavioral disturbances related to the disorder [15]. The role of allopregnanolone in autism is still not very clear and overall, the results on the levels of neurosteroids and related steroids in the body fluids of autistic subjects reported in the literature are poorly studied [16].

Given the complexity of the autism, the molecular mechanism remains unclear, which limits the available therapeutic options. One such approach is dietary supplementation with docosahexaenoic acid (DHA), which has been widely studied for its potential benefits in ASD [17]. Indeed, DHA, a polyunsaturated fatty acid, is considered a serum biomarker for autism, as lower levels of DHA are associated with the diagnosis of ASD [[18], [19], [20], [21]]. DHA is molecule that represents approximately 50–60 ​% of the lipid content of the brain and plays a critical role in various physiological processes throughout the body, including brain function [22,23]. Moreover, DHA has a key role in multiple human organ systems [24,25] and is also a precursor to the specialized pro-resolving lipid mediators (SPMs), which have anti-inflammatory activity and contribute to the inflammation resolution [26]. DHA primarily acts through G-protein–coupled receptor 120 (GPR120/FFAR4) and PPAR-γ, pathways known to suppress inflammatory cytokine production and promote neuronal survival [27]. Among the proposed mechanisms, DHA is also an agonist for peroxisome proliferator-activated receptor type alpha (PPAR-α) and an NF-kB signal inhibitor [28].

To date the relationship between DHA and allopregnanolone levels is not deeply investigated, however a previous preliminary study suggested that there may be an association between lower plasma essential fatty acid status and elevated neurosteroid concentrations in male psychiatric patients with DSM-III alcoholism or depression with several limitations and confounding variables that make necessary future investigations [29].

Another fatty acid amide, that interacts with PPAR-α is palmitoylethanolamide (PEA). Clinical studies indicate that PEA, whether singly or associated with antioxidant or anti-inflammatory compounds, is potentially useful in a wide range of therapeutic areas, including pain management, neurodegeneration, and even the autism spectrum disorder [[30], [31], [32]]. Previous studies have already highlighted the potential effect of the ultramicronized form of PEA (PEA-um) and its associations to improve autistic behaviors, in both mouse models and clinical studies or case reports [33,34]. In particular, we have previously shown the ability of intraperitoneal-injected PEA-um (10 ​mg/kg and 30 ​mg/kg) to revert behavioral phenotype alterations of BTBR mice [35]. Furthermore, recently a new study suggests that PEA may increase the therapeutic effects of risperidone on autism-related irritability and hyperactivity [36]. Its efficacy seems to be correlated primarily through the reduction of neuroinflammation, with the PPAR-α receptor playing a crucial role in this effect [35]. In addition, PEA, acting as a ligand of PPAR-α, might regulate neurosteroidogenesis, inducing peripheral biosynthesis of allopregnanolone [37,38].

Besides neurosteroid dysregulation, several other mechanisms have been incriminated in ASD. Numerous studies have highlighted the presence of immune dysfunctions in individuals with ASD, such as increased activation of both microglia and astroglia, elevated levels of pro-inflammatory cytokines such as interferon (IFN)-γ, interleukin (IL)-1β, IL-6, IL-12, tumor necrosis factor (TNF)-α, and macrophage chemotactic protein (MCP)-1 in brain tissue and cerebrospinal fluid [[39], [40], [41]].

Existing evidence points toward the inflammasomes as key initiators of inflammation [42]. Among them, the NOD-like receptor 3 (NLRP3) inflammasome is particularly relevant due to its central role in IL-1β production and the intensification of neuroinflammatory responses, interfering with normal brain development [43]. Consistent with this, children with ASD show elevated levels of inflammasome-related cytokines, suggesting an enhanced systemic inflammatory state [44], although direct evidence of inflammasome activation in ASD is insufficient [45]. However, the mechanism underlying the hyperactivation of the NLRP3 inflammasome may be linked to oxidative stress and mitochondrial dysfunction, both of which are present in ASD [46,47]. Furthermore, neuroinflammation is known to impair both the expression and signaling of brain-derived neurotrophic factor (BDNF), negatively affecting synaptic plasticity and cognitive function [48]. Interestingly, DHA supplementation appears to modulate synaptic plasticity and cognitive function by acting on BDNF levels [49].

On the basis of this evidence in this study we investigated the role of neurosteroids in ASD using BTBR mice, an inbred strain widely recognized as a model for studying autism, moreover, we studied the role of PPAR-α by the co-administration of PEA-um and DHA in a very low dosage, focusing on the crosstalk between neuroinflammation and neurosteroids in autism.

## Materials and Methods

### Animals

BTBR T ​+ ​tf/J (BTBR), B6.129S4-SvJae-Ppara^tm1Gonz^ PPAR-α null (KO) and their control C57Bl/6J (B6) mice colonies were assessed in our animal facility, genotyped according to Jackson Laboratories supplier webpage, using the RedExtract kit (Sigma–Aldrich, Italy). The decision to use 3-month-old male animals is intentional, as our experimental design focuses on a single period of development (young adulthood) in order to reduce biological variability and specifically evaluate the functional and molecular outcomes of treatment at steady state. In fact, our primary objective is to establish conceptual validity evidence of the efficacy and mechanism of treatment under stable neurobiological conditions, rather than to evaluate developmental trajectories.

Mice were housed in groups in the same room under controlled temperature, humidity, on a 12 ​h:12 ​h light:dark cycle, with ad libitum access to water and standard laboratory chow diet.

All experimental procedures were conducted in conformity with international and national law and policies (EU Directive 2010/63/EU for animal experiments, ARRIVE guidelines and the Basel declaration including the 3R concept) and approved by the Institutional Committee on the Ethics of Animal Experiments (CSV) of the University of Naples Federico II and by the Italian Ministry of Health under protocol no. 354/2022-PR. Every effort was made to restrict the use of animals to as few as possible in our experiments.

### Drugs and treatment

Ultra-micronised PEA (PEAum®; Epitech Group, Italy), with average particle size between 0.2 ​μm and 10 ​μm, and DHA (Cayman Chemical Co.) were dissolved in 1.5 ​% (w/v) carboxymethylcellulose in saline.

PPAR-α antagonist, GW6471 (N-((2S)-2-(((1Z)-1-Methyl-3-oxo-3-(4-(trifluoromethyl) phenyl)prop-1-enyl) amino)-3-(4-(2-(5-methyl-2-phenyl-1,3-oxazol-4-yl)ethoxy)phenyl)propyl; Tocris, Bristol, UK) propanamide) and PPAR-γ antagonist, GW9662 (2-Chloro-5-nitro-N-phenylbenzamide; Tocris, Bristol, UK) were dissolved in PEG400, Tween 80 and sterile saline (Sigma-Aldrich, Milan, Italy) to obtain a final concentration of PEG400 and Tween 80 of 20 and 10 ​% v/v.

BTBR, KO and control B6 mice received orally for 10 days Vehicle, PEA-um 1 ​mg/kg, and/or DHA 5 ​mg/kg and/or GW6471 1 ​mg/kg and/or GW9662 1 ​mg/kg. These doses were selected based on our previous studies proving efficacy in similar models in mice [35,50,51]. When BTBR mice were co-treated with PEA-um and DHA, both drugs were given together in a single administration, while when BTBR mice were co-treated with PEA-um ​+ ​DHA and one of the PPARs antagonists, GW6471 or GW9662, the antagonist was injected 30 ​min before the administration PEA-um ​+ ​DHA.

### Behavioral tests

Behavioral tasks described below were conducted in the same mice from 3 different litters, in a battery on two separate days (day 8 and day 9) according to *Paylor* et al. *2006* [52], with sufficient intervals between tests, in a sequence that begins with the least stressful quick observational test followed by the more stressful complex tasks. All experiments and data analyses were performed by operators blinded to genotype, when possible, and treatment. Behavioral tasks including marble burying (MBT) and three chambered social (TST) tests were performed on day 8, whileself-grooming (SGT) and reciprocal social interaction test (RSI) were performed on day 9. Mice were euthanized on day 10, 1 ​h after the last administration, as illustrated in Fig. 3A. Manual scoring with a stopwatch (for MBT, RSI and SGT) was performed by a trained observer blind to treatment, while automatic scoring (TST) was performed using the AnyMaze video tracking system.

#### Marble burying test

Each mouse was allocated in the test plastic container with 20 glass marbles of 1.5 ​cm in diameter placed on top of 3 ​cm of clean woodchip bedding, arranged in five rows of four and allowed to freely explore the cage for 15 ​min. After the test, each mouse was removed and replaced to its home cage, marbles were cleaned and new bedding placed. When a threshold of 75 ​% coverage for each marble was observed, it was considered buried and recorded.

#### Spontaneous self-grooming behavior

During this test mice were individually allocated in an empty plastic cage and allowed to freely explore the arena for a total time of 20 ​min. After habituation period (10 ​min), time spent in self-grooming was manual scored by a trained observer. Self-grooming behavior included head washing, body grooming, genital/tail grooming and paw and leg licking. After the test, the cage was thoroughly cleaned.

#### Three chambered social test

Social approach behavior was tested in a three-chambered apparatus. as After 5-min habituation phase, a novel sex, strain and age matched mouse is placed in one side of the chamber under an enclose cup, with the other side containing an empty cup. During the sociability phase, the tendency to approach a novel mouse is compared with tendency to approach a novel object. This phase is monitored and recorded for 10 ​min by a video camera coupled with a video-tracking software (Any-maze, Stoelting). Both sides were alternated between the left and right chambers across subjects. The time spent in each chamber following habituation period was analyzed.

#### Reciprocal social interaction test

During the reciprocal social interaction test, we investigated mice interaction. After a 5-min habituation period in the test chamber, the interactions between the mouse tested and a novel sex, strain and age matched mouse were recorded for 20 ​min. The following pro-social interactions were evaluated: number of followings, push-crawl behavior, nose-to-nose and anogenital sniffing, and time spent in self-grooming. Data were manually scored.

### Samples collection and molecular analysis

The day after behavioral tests, mice were anesthetized with alphaxalone (70 ​mg/kg) and acepromazine (10 ​mg/kg) intraperitoneally for blood collection. Blood was collected via cardiac puncture. Then, prefrontal cortex and hippocampus were collected and immediately frozen at −80 ​°C, until use for *ex vivo* analysis.

#### Western Blot analysis

Cortex and hippocampus tissue were homogenized on ice-cold lysis buffer (10 ​mM Tris–HCl, 20 ​mM, pH 7.5, 10 ​mM NaF, 150 ​mM NaCl, 1 ​% Nonidet P-40, 1 ​mM phenyl-methylsulphonylfluoride, 1 ​mM Na3VO4, leupeptin, and trypsin inhibitor 10 ​μg/ml) and total protein lysates underwent SDS-PAGE. Specific binding sites were blocked with 3 ​% non-fat dried milk (Bio-Rad, Hercules, CA, USA) for 45 ​min at room temperature, and incubated at 4 ​°C, overnight in the presence of primary antibodies in the same blocking solution. Subsequently, the filters were incubated with the appropriate secondary antibody (Jackson ImmunoResearch, West Grove, PA, USA) for 1 ​h at room temperature. Bands were detected by ChemiDoc imaging instrument (Bio-Rad, Segrate, Italy). The antibodies used in this manuscript are reported in Supplementary Table S1.

#### Central neurosteroid extraction and measurements

Cortex and hippocampus (∼30 ​mg of tissue) were extracted and homogenated with 150 ​μL of acetonitrile. After a centrifugation, 300 ​μl of hexane was added to the supernatant and shacked to isolate the neurosteroid fraction from the remaining components of the brain tissue. Then, the hexane layer was discarded while the lower heavier layer of acetonitrile evaporated under SpeedVac. The dried steroid residues were resuspended in a solution of EtOH 5 ​%. The amount of allopregnanolone and pregnenolone were determined through mass spectrometry for plasma samples and using commercially available enzyme-linked immune assay kit for cortex and hippocampus (ELISA, Arbor Assays Cat# K061–H1; Mybiosource Cat# MBS3806492) according to the manufacturer's instructions. The data were normalized to the wet tissue weight.

#### Real-time PCR analysis

Total RNA was extracted from prefrontal cortex and hippocampus using Trizol (Ambion). Two micrograms of total RNA were used in first-strand cDNA synthesis (Promega, Madison, WI) according to the manufacturer's instructions. PCRs were performed with a Bio-Rad CFX96 Connect Real-time PCR System instrument and software (Bio-Rad Laboratories). The PCR conditions were 15 ​min at 95 ​°C followed by 40 cycles of two-step PCR denaturation at 94 ​°C for 15 ​s, annealing at 55 ​°C for 30 ​s and extension at 72 ​°C for 30 ​s. Each sample contained 25 ​ng cDNA in 2X QuantiTech SYBRGreen PCR Master Mix and primers *SRD5A1, Akr1c4, Ppara, Bdnf, Ntrk2*, *Tnfa*, *Il1b*, *Il6, ccl2, Nlrp3* and *Tgf1*. The relative amount of each studied mRNA was normalized to GAPDH as housekeeping gene, and the data were analyzed according to the 2^–ΔΔCT^ method. Primer sequences are included in Supplementary Table S2.

#### Plasmatic parameters

Plasma was obtained by blood centrifugation. Plasmatic levels of TNF-α*,* IL-1β and IL-6 were measured using commercially available ELISA kits (MyBioSource Cat# MBS161138, MBS2021142, MBS163272), following the manufacturer's instructions.

### Statistical analysis

Data were expressed as mean ​± ​standard error of the mean (SEM) and analyzed with GraphPad Prism® 10 software (GraphPad Software Inc., San Diego, CA, USA). The sample size for each experiment was determined based on ensuring statistical significance. The Shapiro-Wilk test was used to assess normality distribution. Since all data followed a Gaussian normal distribution, parametric tests were applied, i.e., for analysis of multiple groups, a two-way ANOVA was adopted, followed by Bonferroni's post-hoc multiple comparisons analysis. For analysis of chamber time and sniffing time in TST, a paired two-tailed Student's *t*-test was adopted. A p ​< ​0.05 was considered statistically significant.

## Results

### Low-dose PEA-um ​+ ​DHA elevates systemic and central neurosteroid levels in BTBR mice

Systemic and central concentration of pregnenolone and allopregnanolone was measured through mass spectrometry and ELISA assay, respectively. Single oral treatment with PEA-um (1 ​mg/kg), as well as with DHA (5 ​mg/kg) for 10 days, did not change pregnenolone or allopregnanolone concentration in both B6 and BTBR mice (Supplementary Fig. 1).

**Fig. 1:**
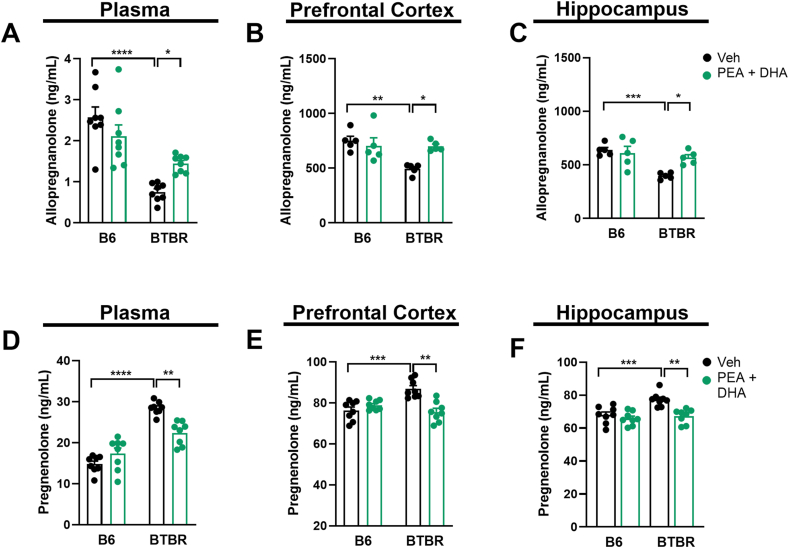
Neurosteroid levels in plasma and brain areas after PEA-um ​+ ​DHA co-treatment. (A) Plasmatic, (B) cortical, and (C) hippocampal level of allopregnanolone in B6 and BTBR mice treated with PEA-um ​+ ​DHA. (D) Plasmatic, (E) cortical, and (F) hippocampal level of pregnenolone in B6 and BTBR mice treated with PEA-um ​+ ​DHA. Data are presented as means ​± ​S.E.M of n ​= ​5–8 mice/group. Statistical analysis was conducted by two-way ANOVA followed by Bonferroni's post hoc-test. ∗∗∗∗p ​< ​0.0001, ∗∗∗p ​< ​0.001, ∗∗p ​< ​0.01 and ∗p ​< ​0.05.

In Veh-BTBR mice, lower plasma allopregnanolone concentration (Fig. 1A) was correlated with the higher level of pregnenolone (Fig. 1D), possibly indicating a feedback mechanism in BTBR mice. PEA-um ​+ ​DHA treatment in BTBR mice significantly increased plasma allopregnanolone (Fig. 1A) and decreased pregnenolone (Fig. 1D) levels, compared to Veh-BTBR mice (p ​< ​0.05, p ​< ​0.01 respectively). Similarly to plasma, brain concentration of allopregnanolone was significantly lower in Veh-BTBR compared to Veh-B6 mice (p ​< ​0.01, p ​< ​0.001 respectively) at both cortical (Fig. 1B) and hippocampal level (Fig. 1C). On the contrary, pregnenolone concentration was higher at both cortical (Fig. 1E) and hippocampal level (Fig. 1F) compared to Veh-B6 mice (p ​< ​0.001). The oral administration of PEA-um ​+ ​DHA significantly increased (p ​< ​0.05) brain allopregnanolone (Fig. 1B–C) and decreased pregnenolone concentration (Fig. 1E–F) compared to Veh-BTBR mice (p ​< ​0.01). In B6 mice, treatment with PEA-um ​+ ​DHA did not significantly change neurosteroid concentration compared to Veh-B6 mice, in any of the analyzed tissues.

### Low-dose PEA-um ​+ ​DHA repristinate the 5α-R1 and 3-α-HSD expression in BTBR mice

**Fig. 2:**
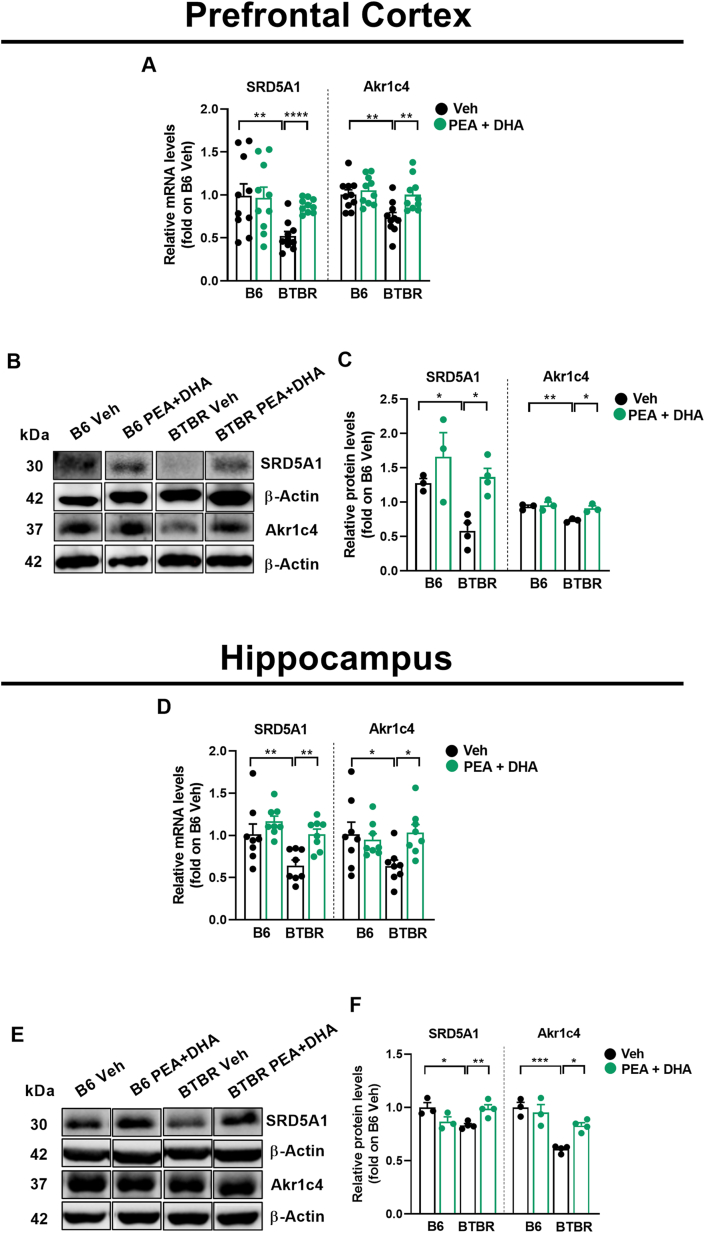
PEA-um ​+ ​DHA co-treatment effect on cortical and hippocampus gene and proteins expression of neurosteroidogenic enzymes in BTBR mice. (A) qPCR showing the changes in mRNA levels (fold of B6+Veh) of 5αR1 (SRD5A1) and 3α-HSD (Akr1c4) in prefrontal cortex. (B) Western blot images for SRD5A1, Akr1c4 and β-actin performed on prefrontal cortex homogenates, representative of n ​= ​3–4 mice/group. (C) Western blotting quantifications (fold of B6+Veh) showing the changes of 5αR1 (SRD5A1) and 3α-HSD (Akr1c4) in prefrontal cortex. (D) qPCR showing the changes in mRNA levels (fold of B6+Veh) of 5αR1 (SRD5A1) and 3α-HSD (Akr1c4) in the hippocampus. (E) Western blot images for SRD5A1, Akr1c4 and β-actin performed on hippocampus homogenates, representative of n ​= ​3–4 mice/group. (F) Western blotting quantifications (fold of B6+Veh) showing the changes of 5αR1 (SRD5A1) and 3α-HSD (Akr1c4) in the hippocampus. Data are presented as means ​± ​S.E.M of (A, D) n ​= ​8–10 mice/group. (F) n ​= ​3–4 mice/group. Statistical analysis was conducted by two-way ANOVA followed by Bonferroni's post hoc-test. ∗∗∗∗p ​< ​0.0001, ∗∗∗p ​< ​0.001, ∗∗p ​< ​0.01 and ∗pnbsp;< ​0.05.

To better understand the mechanism by which PEA-um ​+ ​DHA repristinate allopregnanolone levels, we studied the mRNA and protein expression of two enzymes involved in neurosteroidogenesis, 5αR1 (SRD5A1) and 3α-HSD (Akr1c4) (Fig. 2). The mRNA expression of SRD5A1 was significantly reduced in Veh-BTBR compared to Veh-B6 mice, both at cortical (Fig. 2A; p ​< ​0.01) and hippocampal (Fig. 2D; p ​< ​0.01) levels. Also, the mRNA expression of Akr1c4 was significantly reduced in prefrontal cortex (Fig. 2A; p ​< ​0.01) as in the hippocampus (Fig. 2D; p ​< ​0.05) of Veh-BTBR mice compared to Veh-B6 mice. PEA-um ​+ ​DHA administration normalized the mRNA expression of both enzymes to the levels observed in Veh-B6 mice, in the prefrontal cortex (Fig. 2A; p ​< ​0.0001, p ​< ​0.01). and hippocampus (Fig. 2D; p ​< ​0.01, p ​< ​0.05).

**Fig. 3:**
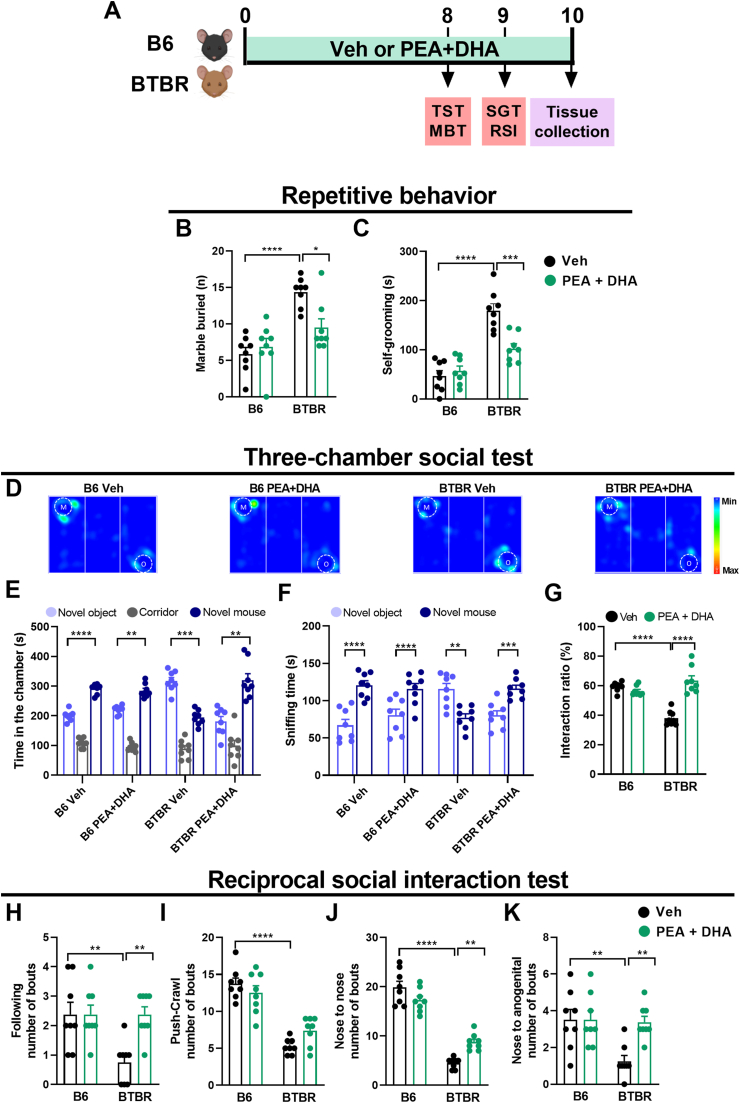
PEA-um ​+ ​DHA attenuated ASD-like behaviors. (A) The experimental schedule of behavioral tests following PEA-um ​+ ​DHA co-treatment. (B) The number of buried marbles in the marble burying test (MBT). (C) Time mice spent grooming during the self-grooming test (SGT). (D) Representative heat maps showing the mice's time exploring in the three-chamber social test (TST). “M” and “O” represent the novel mouse and the novel object, respectively. (E) The amount of time spent in the chambers, (F) sniffing time and (G) preference index of time spent sniffing in the three-chamber social test (TST). Number of (H) following, (I) push and crawl, (J) nose-to-nose and (K) nose-to-anogenital in the reciprocal social interaction (RSI) test. Data are presented as means ​± ​S.E.M of n ​= ​8 mice/group. Statistical analysis was conducted by two-way ANOVA followed by Bonferroni's post hoc-test (B, G-K) or paired t-test (E, F). ∗∗∗∗p ​< ​0.0001, ∗∗∗p ​< ​0.001, ∗∗p ​< ​0.01 and ∗p ​< ​0.05.

Consistently, the protein levels of these two steroidogenic enzymes were significantly downregulated in Veh-BTBR mice, as shown by Western blot results (Fig. 2B–E) and relative quantifications, in both prefrontal cortex (Fig. 2C SRD5A1 p ​< ​0.05 and Akr1c4 p ​< ​0.01) and hippocampus (Fig. 2F SRD5A1 p ​< ​0.05 and Akr1c4 p ​< ​0.001). PEA-um ​+ ​DHA treatment normalized the protein expression to the levels observed in B6-veh mice in both tissues (Fig. 2C both p ​< ​0.05; Fig. 2F SRD5A1 p ​< ​0.01 and Akr1c4 p ​< ​0.05).

### Low dose PEA-um ​+ ​DHA association improved repetitive and social behaviors of BTBR mice

To investigate the effect of combined low-dose PEA-um and DHA administration on ASD-like behaviors, BTBR mice received by oral gavage both PEA-um (1 ​mg/kg) and DHA (5 ​mg/kg) alone and their association for 10 days (Fig. 3A). Single oral treatment with PEA-um, as well as with DHA, did not rescue ASD-like behavior in all behavioral tests of both B6 and BTBR mice (Supplementary Fig. 2).

Marble-burying (MBT) and self-grooming (SGT) tests were used to assess repetitive behaviors. Veh-BTBR mice buried significantly more marbles (Fig. 3B) and exhibited more self-grooming behavior (Fig. 3C) than Veh-B6 mice (p ​< ​0.0001). BTBR mice treated with the low dose PEA-um ​+ ​DHA combination showed a significant reduction of marbles buried (Fig. 3B) and of self-grooming time (Fig. 3C) compared to Veh-BTBR mice (p ​< ​0.05, p ​< ​0.001 respectively). In B6 mice, PEA-um ​+ ​DHA did not show any significant effect.

In addition, social behavior was evaluated in two behavioral tasks: the three chambered social test (TST) and the reciprocal social interaction (RSI). In the three-chambered social test, B6 mice spent more time in the chamber with a mouse (Fig. 3D–E) and more time in sniffing (Fig. 3D–F) the mouse, than the object (p ​< ​0.0001), while Veh-BTBR mice preferred to spend more time in the chamber with the object and in sniffing the object (p ​< ​0.001, p ​< ​0.01 respectively). The oral administration of the low-dose PEA-um ​+ ​DHA combination to BTBR mice significantly increased time spent in social interaction (Fig. 3E) and sniffing time (Fig. 3F) in the mouse side of the apparatus (p ​< ​0.01 and p ​< ​0.001 respectively). The interaction ratio (Fig. 3G) revealed a significant effect of the PEA-um ​+ ​DHA treatment in BTBR mice (p ​< ​0.0001). On the contrary, the tested combination did not have any impact on B6 pro-social attitude (Fig. 3D–G). Mice were also tested in the reciprocal social interaction test (Fig. 3H–K). Different parameters were considered: number of followings, push-crawl, nose to nose and nose-anogenital sniffing. As expected, all parameters were significantly decreased in BTBR mice compared to B6 mice (p ​< ​0.01, p ​< ​0.0001, p ​< ​0.0001, p ​< ​0.01 respectively). The oral administration of low-dose PEA-um ​+ ​DHA improved the social phenotype of BTBR mice, significantly increasing the number of followings (Fig. 3H), the number of nose-to-nose (Fig. 3J) as well as nose-anogenital sniffing (Fig. 3K), compared to Veh-BTBR mice (p ​< ​0.01).

### Role of PPARs in the mechanism of action of low-dose PEA-um ​+ ​DHA association on behavioral phenotype of BTBR mice

**Fig. 4:**
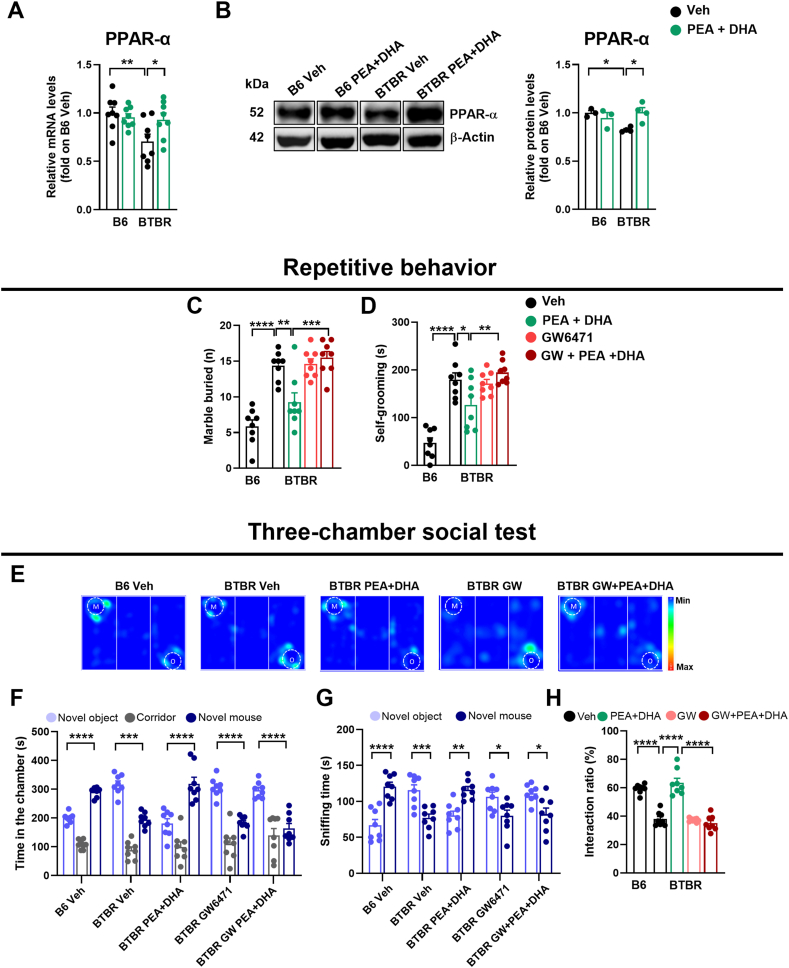
Role of PPAR-α in the mechanism of action of PEA-um ​+ ​DHA association. (A) qPCR showing the changes in mRNA levels (fold of B6+Veh) of peroxisome proliferator-activated receptor (PPAR)-α in the hippocampus. (B) Western blot images for PPAR-α and β-actin performed on hippocampus homogenates, representative of n ​= ​3–4 mice/group, and quantifications (fold of B6+Veh) showing the changes of PPAR-α in the hippocampus. (C) The number of buried marbles in the marble burying test. (D) Time mice spent grooming during the self-grooming test. (E) A representative heath maps of the three-chamber social test for each group. “M” and “O” represent the novel mouse and the novel object, respectively (F) The amount of time spent in the chambers, (G) sniffing time and (H) preference index of time spent sniffing in the three-chamber social test (TST). Data are presented as means ​± ​S.E.M of (A, C, D, F, G, H) n ​= ​8 mice/group and (B) n ​= ​3–4 mice/group. Statistical analysis was conducted by one or two-way ANOVA with Bonferroni's post-hoc analysis (A-D, H) or paired t-test (F, G). ∗∗∗∗p ​< ​0.0001, ∗∗∗p ​< ​0.001, ∗∗p ​< ​0.01 and ∗p ​< ​0.05.

To assess the involvement of PPARs in the mechanism of action of PEA-um and DHA, in vivo and ex-vivo experiments were performed. We first evaluated the role of PPAR-α in the hippocampus, since it is known that BTBR mice displayed a significant reduction in PPAR-α mRNA and protein expression compared to B6 mice [35]. Our data confirm the down regulation of PPAR-α mRNA and protein expression in the hippocampus of BTBR mice (Fig. 4A–B; p ​< ​0.05 and p ​< ​0.01, respectively), and showed that low-dose of PEA-um associated with low-dose of DHA were able to restore both PPAR-α mRNA (Fig. 4A; p ​< ​0.05) and protein (Fig. 4B; p ​< ​0.05) hippocampal expressions.

Then, we investigated the role of PPAR-α by behavioral tests, treating BTBR mice with the PPAR-α antagonist GW6471 (1 ​mg/kg) before PEA-um ​+ ​DHA administration. In marble burying (Fig. 4C), self-grooming (Fig. 4D) and three-chambered social tests (Fig. 4E–H), GW6471 significantly inhibited PEA-um ​+ ​DHA effects (from p ​< ​0.05 to p ​< ​0.0001 respectively).

Finally, by in vivo experiments, we also investigated the role of PPAR-γ using a selective antagonist, GW9662. Behavioral results showed that GW9662 (1 ​mg/kg) had a weak and non-significant effect on PEA-um ​+ ​DHA activity in BTBR mice (Supplementary Fig. 3). Based on these results, we did not perform *ex vivo* analysis.

### Low dose-PEA-um ​+ ​DHA association attenuates inflammasome up-regulation in BTBR mice

**Fig. 5:**
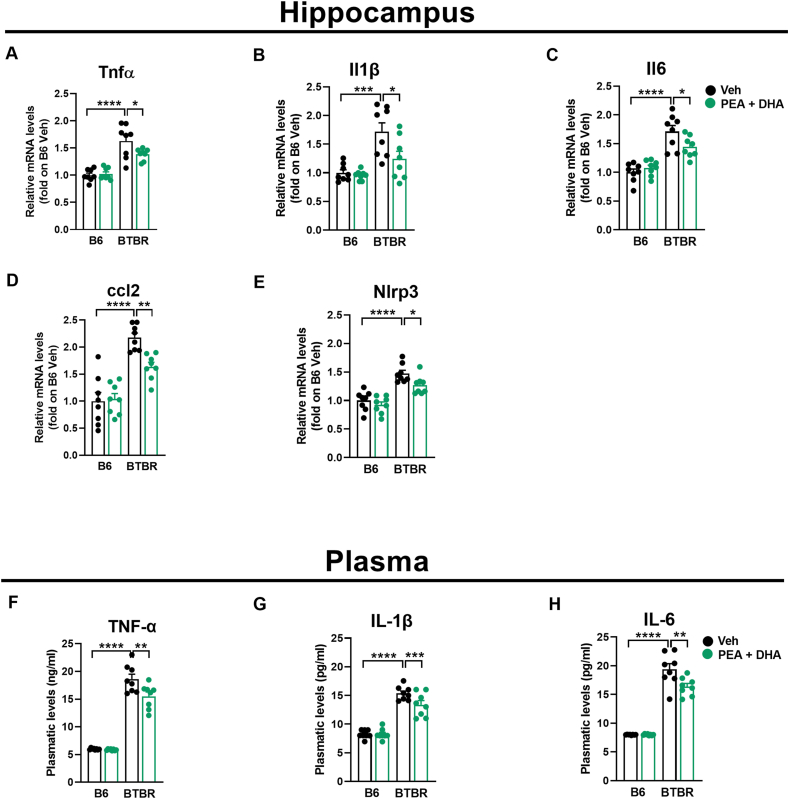
PEA-um ​+ ​DHA modulate hippocampal and plasmatic inflammatory cytokines. Changes in (A) Tnf-α, (B) IL-1β, (C) IL-6, (D) ccl-2 and (E) Nlrp3 mRNA levels in hippocampus, and cytokine analysis of (F) Tnf-α, (G) IL-1β, (H) IL-6 in plasma. Data are presented as means ​± ​S.E.M of n ​= ​8 mice/group. Statistical analysis was conducted by two-way ANOVA with Bonferoni's post-hoc analysis. ∗∗∗∗p ​< ​0.0001, ∗∗∗p ​< ​0.001, ∗∗p ​< ​0.01 and ∗p ​< ​0.05.

Central and systemic effects of PEA-um ​+ ​DHA association on proinflammatory cytokines, chemokines, and the involvement of the NLRP3 inflammasome activation were then assessed in BTBR mice (Fig. 5).

The significant increased levels of Tnfα (p ​< ​0.0001), Il1β (p ​< ​0.001), Il6 (p ​< ​0.0001) and ccl2 (p ​< ​0.0001) mRNA observed in the hippocampus of Veh-BTBR mice compared to Veh-B6 mice were significantly reduced (p ​< ​0.05 and for ccl2 p ​< ​0.01) by PEA-um ​+ ​DHA association (Fig. 5A- D).

Moreover, we evaluated the involvement of inflammasome in the regulation of proinflammatory cytokines by PEA-um ​+ ​DHA combination. Nlrp3 mRNA expression was significantly increased (p ​< ​0.0001) in Veh-BTBR mice and PEA-um ​+ ​DHA association significantly decreased Nlrp3 mRNA expression (Fig. 5E; p ​< ​0.05).

Similarly, the significant increase (p ​< ​0.0001) in plasma cytokines (TNF-α, IL-1β, IL-6) in Veh-BTBR compared to Veh-B6 mice were also significantly reduced (p ​< ​0.01, p ​< ​0.001, p ​< ​0.01 respectively) by PEA-um ​+ ​DHA association (Fig.5D-E-F).

### Low-dose PEA-um ​+ ​DHA association modulates brain-derived neurotrophic factor

**Fig. 6:**
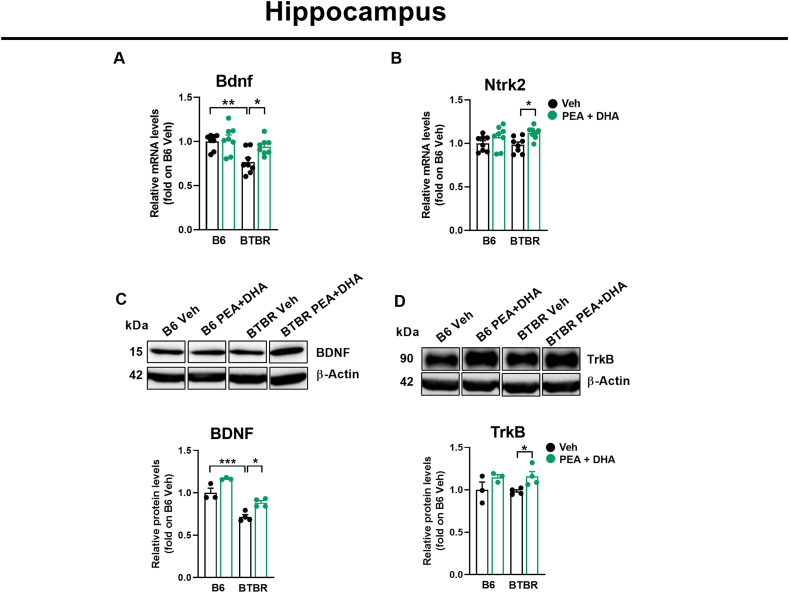
PEA-um ​+ ​DHA induces BDNF/TrkB system at hippocampal level. (A, B) qPCR showing the changes in mRNA levels (fold of B6+Veh) of Bdnf and Ntrk2 in the hippocampus. (C, D) Western blot images for BDNF, TrkB and β-actin, representative of n ​= ​3–4 mice/group, and quantifications (fold of B6+Veh) showing the changes of BDNF and TrkB in the hippocampus. Data are presented as means ​± ​S.E.M of n ​= ​8 mice per group. Statistical analysis was conducted by two-way ANOVA with Bonferroni's post-hoc analysis. ∗∗∗p ​< ​0.001, ∗∗p ​< ​0.01 and ∗p ​< ​0.05.

Our findings confirm prior reports [35] of a downregulation of Bdnf mRNA (Fig. 6A; p ​< ​0.01) and protein expression (Fig. 6C; p ​< ​0.001) in Veh-BTBR mice compared to control B6 mice. PEA-um ​+ ​DHA association significantly increased mRNA and protein expression (p ​< ​0.05) of BDNF in the hippocampus of BTBR mice (Fig. 6A–C). No changes were observed in the mRNA and protein expression of its receptor TrkB (Fig. 6B–D) in Veh-BTBR mice compared to control B6 mice. Following PEA-um ​+ ​DHA administration, a significant increase of TrkB at mRNA and protein expression level were observed (Ntrk2, Fig. 6B, p ​< ​0.05).

## Discussion

The present findings indicate that autism is associated with alterations in neurosteroid synthesis and concentrations, and that the combined administration of low-dose ultramicronized palmitoylethanolamide (PEA-um, 1 ​mg/kg) and docosahexaenoic acid (DHA, 5 ​mg/kg) could restore the physiological condition. Interestingly, at these low doses, neither PEA-um nor DHA alone produced any significant molecular and behavioral effects, suggesting that the observed outcomes may result from a synergistic interaction between the two compounds. Specifically, we observed a significant reduction in allopregnanolone levels in both central nervous system and plasma of BTBR mice, which was shown to depend upon the decreased activity of the steroidogenic enzymes responsible for its synthesis. Co-administration of low-dose PEA-um and DHA reactivated this enzymatic pathway restoring neurosteroid concentrations. Finally, we demonstrated the key role of PPAR-α in PEA ​+ ​DHA activity, and that the co-administration was also able to reduce neuroinflammation process.Neurosteroid are synthesized in both glial cells and neurons in various brain regions, such as the hippocampus and prefrontal cortex. Neurosteroids, like allopregnanolone, are known to be as endogenous modulators of GABA-A receptors [53], which are responsible for excitatory and inhibitory transmission. The mechanism by which neurosteroids modulate the GABA-A receptor has sparked interest in developing new potential therapies for CNS disorders. Studies have shown that BTBR mice present dysregulation of excitatory and inhibitory signaling in different brain areas, due to increased glutamatergic (excitatory) transmission or reduced GABAergic (inhibitory) signaling [14]. In some CNS pathological conditions, the enzymatic activity involved in neurosteroid synthesis is altered and has been associated with behavioral symptoms. For example, *Ebihara* et al. [54] demonstrated that SKF, a 5α-reductase inhibitor, induced sociability deficits, suggesting that low allopregnanolone levels in the brain may contribute to core symptoms of ASD due to impairments in its biosynthesis. In addition, *Kratsman* et al., showed that a SCFA, sodium butyrate, another PPAR-α agonist, attenuates social behavior deficits in BTBR mice, through modulation of the transcription of inhibitory/excitatory genes in the frontal cortex [55].

In line with these findings, here we report for the first time that in BTBR mice, the enzymes 5αR1 and 3α-HSD are downregulated, leading to altered concentrations of allopregnanolone and pregnenolone, and that co-administration of PEA-um and DHA can restore these neurosteroid levels through PPAR-α mediated mechanisms. Our group were among the pioneers in exploring the relationship between PEA and neurosteroids, with a particular focus on the potential of this molecule to modulate GABA-A receptors activity and promote the biosynthesis of allopregnanolone. In 2010 and in 2012 [56], [57], we showed that PEA, via a PPAR-α-mediated pathway, contributes to the synthesis of neurosteroids by elevating their concentrations, in particular allopregnanolone. In these studies, we investigated in Swiss mice, how PEA influences the expression of StAR and P450scc, two proteins involved in the initial stages of neurosteroids production in the brainstem. In these cases, elevated levels of allopregnanolone have a beneficial impact on alertness, consciousness and pain perception. In agreement with our results, in mouse models subjected to stress, decreased levels of allopregnanolone in corticolimbic neurons have been linked to anxiety-like behaviors and heightened fear responses [58]. Furthermore, supplementation with allopregnanolone or treatment with neurosteroidogenic agents, such as PPAR-α agonists, has been shown to alleviate these behavioral deficits [37].

In this study, we show for the first time that ASD is correlate with alteration in the neurosteroidogenesis, in fact, in BTBR mice allopregnanolone concentration in serum and in CNS, is lower with respect to control mice. This condition may contribute to both the development and the progression of autism symptoms, and as we have demonstrated, PPAR- α plays an important role in this process. ASD is defined by deficits in social communication, repetitive behaviors, and restricted interests [59]. Here, we demonstrate that the co-administration of PEA-um and DHA improves social behaviors in individuals with ASD, as evidenced by results from the marble test and the three-chambered social test (TST), as well as from reciprocal social interaction (RSI) and self-grooming behaviors. PEA exerts its effects through multiple mechanisms, numerous studies have established that its primary mode of action involves the activation of PPAR-α, mediating a spectrum of anti-inflammatory, neuroprotective, and neurosteroidogenic effects [60]. This receptor is broadly expressed in several regions of the central nervous system, including microglia, hippocampus, and prefrontal cortex, and plays a key role in maintaining lipid homeostasis and regulating inflammatory responses, also in ASD [61]. Similarly, although it is not its primary mechanism of action, DHA is also able to operates through a PPAR-α mediated mechanism in different pathological conditions [[62], [63], [64], [65]]. The PPAR-α activation leads to a cascade of intracellular events including the reduction of different pro-inflammatory cytokines and the increase of the expression of brain-derived neurotrophic factor (BDNF), a key regulator of neuronal survival and plasticity. Based on this understanding, we hypothesized that the co-administration of low doses PEA-um and DHA could enhance their effects compared to individual treatments. Recently, *Pinna* et al., [66] highlighted that impaired PPAR-α function can lead to reduced neurosteroidogenesis, potentially resulting in behavioral phenotypes resembling symptoms of anxiety and depression in humans. Our findings are in line with these findings, which showed that low-dose PEA-um associated with DHA was able to restore PPAR-α expression in the central nervous system. Moreover, this hypothesis is further supported by considering that co-administration PEA-um with DHA failed to exert effects on the repetitive behavior model when a selective PPAR-α antagonist (GW6471) was used. This is surprising, as both compounds are PPAR-α agonists and one might have expected mutual inhibition. However, this was not observed; instead, a synergistic effect between the two compounds emerged. This finding raises the possibility that PEA-um and DHA can bind two distinct regions of the PPARα-ligand-binding domain, as suggested by a previous crystallographic study [67], contributing to our understanding of the complex relationship between PEA, DHA and neurosteroid synthesis and the potential beneficial implications for these molecules in the regulation of neuropsychiatric disorders.

To support our theory, several studies have shown that PEA activates PPAR-α in cell models and in vivo [68,69], and structural analyses have established that the PPAR-α ligand binding domain (LBD) can accommodate a broad spectrum of fatty acid-derived ligands through distinct pocket regions, supporting the possibility that chemically diverse molecules such as PEA and DHA may interact differently with the receptor [62,67]. Furthermore, some DHA-derived metabolites (e.g., 7(S)-HDHA) have been shown to bind and activate PPAR-α, strengthening the mechanistic plausibility of DHA-related activation [70].

Our study could not overlook a potential role of the PPAR-γ, for two reasons: (i) DHA is an agonist of this receptor; (ii) it is possible a cross-talk between PPAR-α and PPAR-γ. Several studies show that the anti-inflammatory activity of PPAR-γ agonists is related to a significant improvement in CNS disorders, such as depression, anxiety, or neurodegenerative diseases such as Alzheimer's [71]. DHA, through PPARs heterodimers, is involved in regulating BDNF activation [72], and plays an important role in cell function and the development of normal brain cognitive functions, including learning and memory [73]. In our study, the role of PPAR-γ does not appear to be decisive, as the antagonist GW9662 was unable to significantly abolish the effect of PEA ​+ ​DHA on animal behavior, possibly due to the low dosage used. Finally, our data cannot exclude the probable of cross-talk between the two PPAR isoforms; Coactivators, coregulators and cofactors such as RXR and PGC-1α, can participate in both PPARs pathways. It is possible that, under our experimental conditions, the interconnecting pathways were less effective or at least not fully active, and this could explain the weak and non-significant effect of GW9662. Further studies will be necessary to better evaluate this aspect. Furthermore, in several central nervous system disorders, a bidirectional relationship has been found between GABAergic neurotransmission and neuroinflammation, which contribute to the cognitive and motor deficits observed in these CNS disorders [74]. Proinflammatory cytokines and other mediators can directly impact neurons and gliocytes, alter the dendritic spine and neuronal connectivity, and evoke abnormal behaviors [75,76]. In most cases GABAergic signaling has been reported to have anti-inflammatory effects by directly suppressing the functions of certain peripheral immune cells, such as macrophages and lymphocytes. Additionally, PEA and DHA are well-known for their anti-inflammatory properties [77]. Therefore, the combination of PEA-um and DHA, along with the restoration of neurosteroids levels, may contribute to the reduction of the inflammatory state in ASD. Our data indicate that the combination of PEA-um and DHA significantly reduces in the hippocampus of BTBR mice, several pro-inflammatory cytokines, as TNF-α, IL-1β, IL-6, and MCP-1. Notably, IL-1β has been shown to promote neural progenitor cell proliferation in some regions of the CNS, while inhibiting it in others [78]. These region-specific effects may contribute to the patterns of localized over and under growth observed in the brains of individuals with ASD. The formation of excitatory synapses is partly mediated by the IL-1 receptor and associated proteins [79]. In addition, IL-1β enhances the balance between excitatory to inhibitory synapses in cerebellar granule cell cultures [80]. This finding is particularly relevant in the context of autism, as an imbalance between excitatory and inhibitory synaptic inputs is thought to play a central role in its pathogenesis. Moreover, IL-6 is transcribed in the hippocampus during long-term potentiation (LTP) [81]. While overexpression of IL-6 impairs LTP [82,83] reduced IL-6 expression has been associated with enhanced LTP and improved learning and memory [81,84]. In terms of social behavior, mice overexpressing IL-6 exhibit increased sociability compared to IL-6-deficient mice, which display heightened aggression and emotionality [85,86]. Our results show that the low-dose combination of PEA-um and DHA modulates IL-1β and IL-6 signaling, along with other pro-inflammatory mediators, effectively reducing neuroinflammation in BTBR mice. Since the inflammasome is one of the regulators of pro-inflammatory cytokine synthesis, such as IL-1β and IL-18 [87], which were altered in BTBR mice, we have also investigated its involvement. We also found that the combination of PEA-um ​+ ​DHA reduced the activation of the NOD-like receptor 3 (NLRP3) inflammasome in the hippocampus of BTBR mice. Notably, autistic children have high levels of proinflammatory cytokines related to the inflammasome in their blood, indicating the presence of a strong systemic inflammatory response. Similarly, our results show that in BTBR mice, plasma levels of TNFα, IL-1β, and IL-6 were significantly increased and then reduced following treatment with PEA-um ​+ ​DHA, and this also occurs centrally. These findings underscore the importance of targeting the inflammasome as a valid strategy for managing neuroinflammation in ASD. Furthermore, BDNF appears to be a potential target of DHA, which increases its synthesis through the activation of CREB phosphorylation [88]. Aberrant synaptic development, also observed in ASD, can disrupt the production of growth factors, including those involved in the BDNF signaling pathway [89]. Recent studies have provided compelling evidence supporting the involvement of BDNF in autism through its neurotrophic effects on the developing brain. While some studies report elevated blood levels of BDNF in children with ASD [90], others indicate reduced BDNF levels in autistic patients [[91], [92], [93]] and in the hippocampus of BTBR mice [[94], [95], [96]]. Importantly, both PEA-um and DHA have been shown to increase BDNF levels [35,49]. Moreover, neurosteroidogenesis and BDNF expression are related to each other [97]. Additionally, BDNF levels are negatively correlated with oxidative stress [91] and DHA is known to induce antioxidant enzymes [98], suggesting that increasing of BDNF levels could be a consequence of the induction of antioxidant enzymes by DHA that reduce oxidative stress. Our findings align with this notion, as we observed decreased levels of BDNF and its receptor in BTBR mice, which were restored following low dose PEA-um ​+ ​DHA co-administration in the hippocampus.

Although in the present study we have demonstrated that PEA combined with DHA attenuates autistic behaviors via activation of the PPAR-α receptor, this does not demonstrate that PPAR-α is solely sufficient, and that cross-talk between PPAR subtypes or between nuclear and membrane receptors (e.g., PPAR-γ and GPR120) could contribute to the full biological profile of DHA and PEA. In fact, PPAR-α and PPAR-γ often exhibit functional overlap and cooperative transcriptional regulation in lipid metabolism and inflammation [99]. Moreover, GPR120 activation by DHA can lead to intracellular signaling cascades (β-arrestin2, ERK1/2, AMPK) that ultimately converge on PPAR-regulated gene expression [27]. Thus, we cannot exclude the possibility that these two molecules may also modulate the ASD phenotype through alternative molecular pathways.

The balance between pro-inflammatory and anti-inflammatory mediators, such as resolvins, may significantly influence the severity of the ASD condition, as repeatedly suggested. A recent study, the authors demonstrated that the administration of resolvin D1 reduced microglial activation and neuroinflammation in animal models of neurodevelopmental disorders, indicating that resolvins may hold therapeutic potential for ASD [100]. Since DHA is known to modulate inflammation resolution through the activation of resolvin synthesis [101], it is possible that this pathway, which we did not investigate in this study, may also play an important role. In this case, PEA could act synergistically with DHA by enhancing the anti-inflammatory response. This hypothesis opens new avenues for future studies aimed at clarifying other possible underlying mechanisms involved. Furthermore, both inflammation and the activation of inflammation lead to the development of autism-like behavioral deficits [102] and the same reduction in central BDNF levels caused by pro-inflammatory cytokines can lead to ASD-like symptoms through the compromise of neurogenesis and synaptic plasticity [15].

The key role of PPAR-α in the effects of PEA-um ​+ ​DHA may indicate that its reduction in BTBR mice could underlie an altered response to neurosteroids. All mechanistic hypotheses done open the door for future studies targeted to further explore the potential mechanisms of action of the two molecules used synergistically in this work.

Overall, these results open up new scenario for the management and possible treatment of ASD. Regarding clinical transferability, it is important to note that both PEA and DHA have been studied independently in human trials. For example, PEA has been shown to significantly improve irritability and hyperactivity in children with ASD [36], while another study found that PEA supplementation can improve behavioral symptoms through neuroinflammatory and endocannabinoid modulation [31]. As for DHA, omega-3 supplementation has been examined, finding significant increases in plasma DHA in patients with ASD and trends toward improved adaptive behavior [21,103]. In another study, this supplementation led to a reduction in plasma IL-2 concentrations, suggesting immunomodulatory benefits [104]. Overall, these human studies support the translational plausibility of the mechanisms studied in our BTBR model, particularly those involving neuroinflammation, synaptic function, and lipid signaling, while emphasizing the need for further controlled clinical trials to confirm efficacy.

The concomitant use of low doses of PEA-um and DHA allows for a reduction in the effective dosage required to achieve tangible benefits in terms of both behavior and control of neuroinflammation, through the modulation of neurosteroids, potentially contributing to the development of new therapeutic strategies for ASD that involve the PPAR-α receptor and neurosteroidogenesis in their mechanism of action.

## Author contributions

**Fabiana Filogamo:** Writing – original draft, Investigation, Formal analysis, Methodology. **Fabrizio Maria Liguori:** Investigation, Methodology. **Giovanna La Rana:** Data curation, Software. Roberto Russo: Writing – review & editing, Supervision, Resources, Conceptualization. **Claudia Cristiano:** Writing – original draft, Visualization, Project administration, Resources, Supervision, Conceptualization.

## Data availability

Data will be made available on request.

## Funding

This work was partially supported by a grant assigned to CC from Epitech Group S.p.A. The funding organization had no influence on (1): the study design (2), the collection, analysis, and interpretation of data (3); the writing of the manuscript; and (4) the decision to submit the manuscript for publication.

## Declaration of competing interest

The authors declare the following financial interests/personal relationships which may be considered as potential competing interests:This article has been conducted and written in the absence of any commercial or financial relationships that could be construed as a potential conflict of interest. The author CC is co-inventor of patent for the therapeutic use of PEA and DHA in autism and other diseases. (IT patent no.: 102021000024464). The authors declare that no other conflicts of interest exist.
