# Supplementary material for: Low-dose combination of ultramicronized palmitoylethanolamide and docosahexaenoic acid on neurosteroid and neuroinflammatory dysregulation in autism spectrum disorders
Source: Neurotherapeutics. 2025 Dec 13;23(1):e00816. doi: 10.1016/j.neurot.2025.e00816 (PMC12976511; doi:10.1016/j.neurot.2025.e00816)
Supplement: Multimedia component 1 [file mmc1.docx]

**Supporting Information**

**Low-dose combination of ultramicronized palmitoylethanolamide and docosahexaenoic acid on neurosteroid and neuroinflammatory dysregulation in autism spectrum disorders.**

Fabiana Filogamo^a^, Fabrizio Maria Liguori^a,1^, Giovanna La Rana^a^, Roberto Russo^a*^ Claudia Cristiano^a^.

**Contents:**

Fig. S1. PEAum+DHA elevates systemic and central neurosteroids levels in BTBR mice.

Fig. S2. PEAum+DHA association improved repetitive and social behaviors of BTBR mice.

Fig.S3. Role of PPAR-γ in PEA-um+DHA activity.

Table S1. The antibodies used in this study.

Table S2. The sequences of primers used in this study.


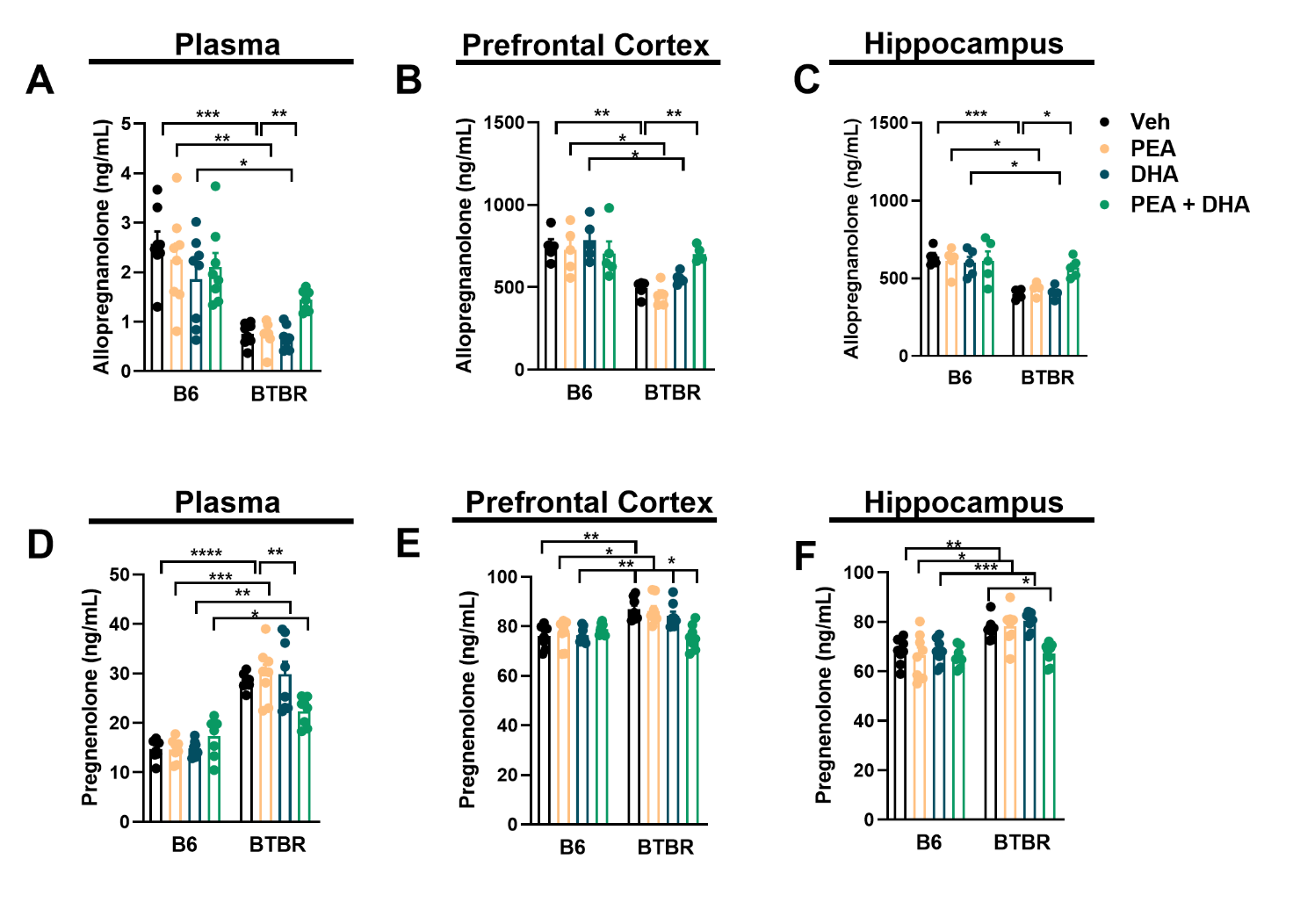


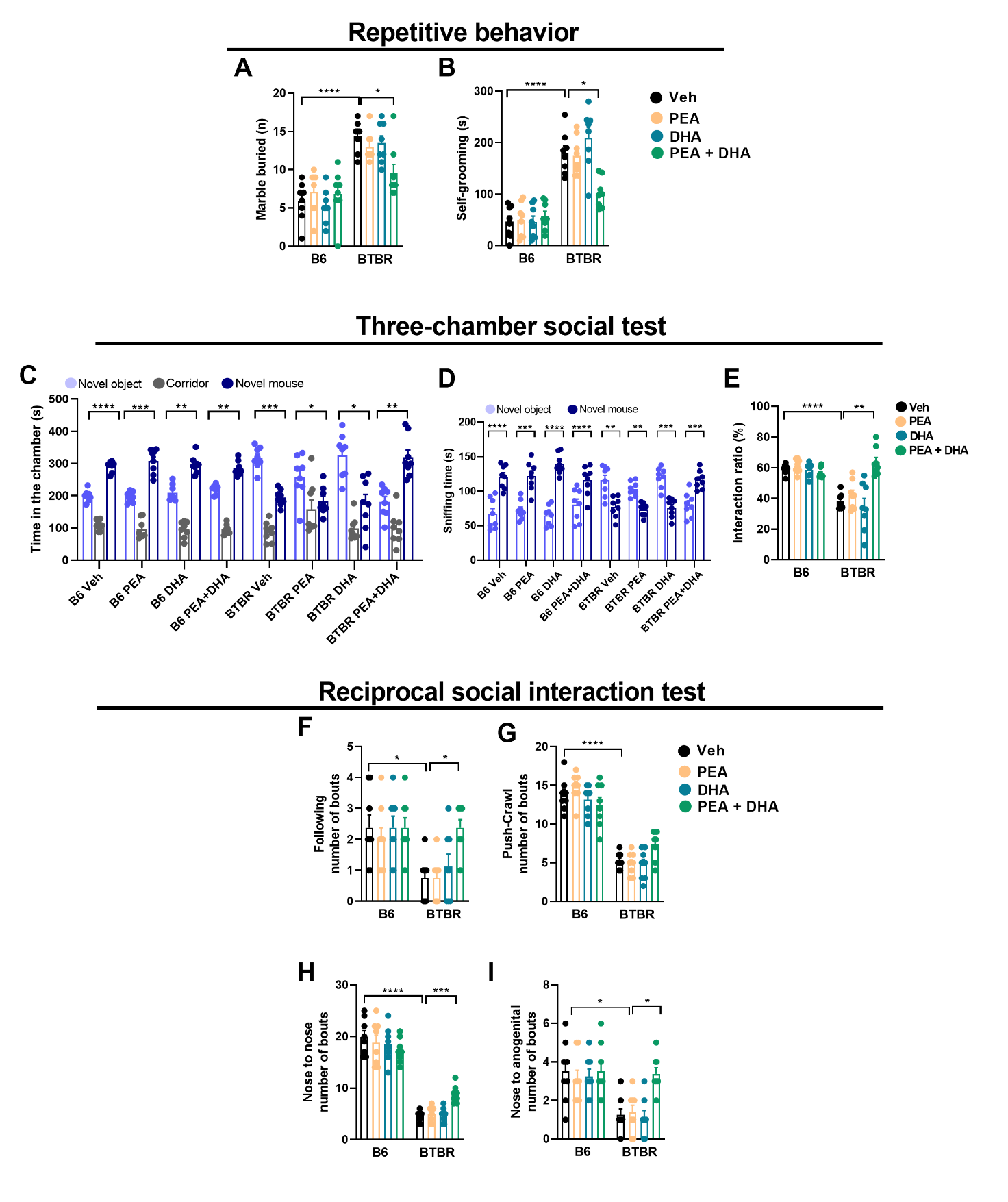


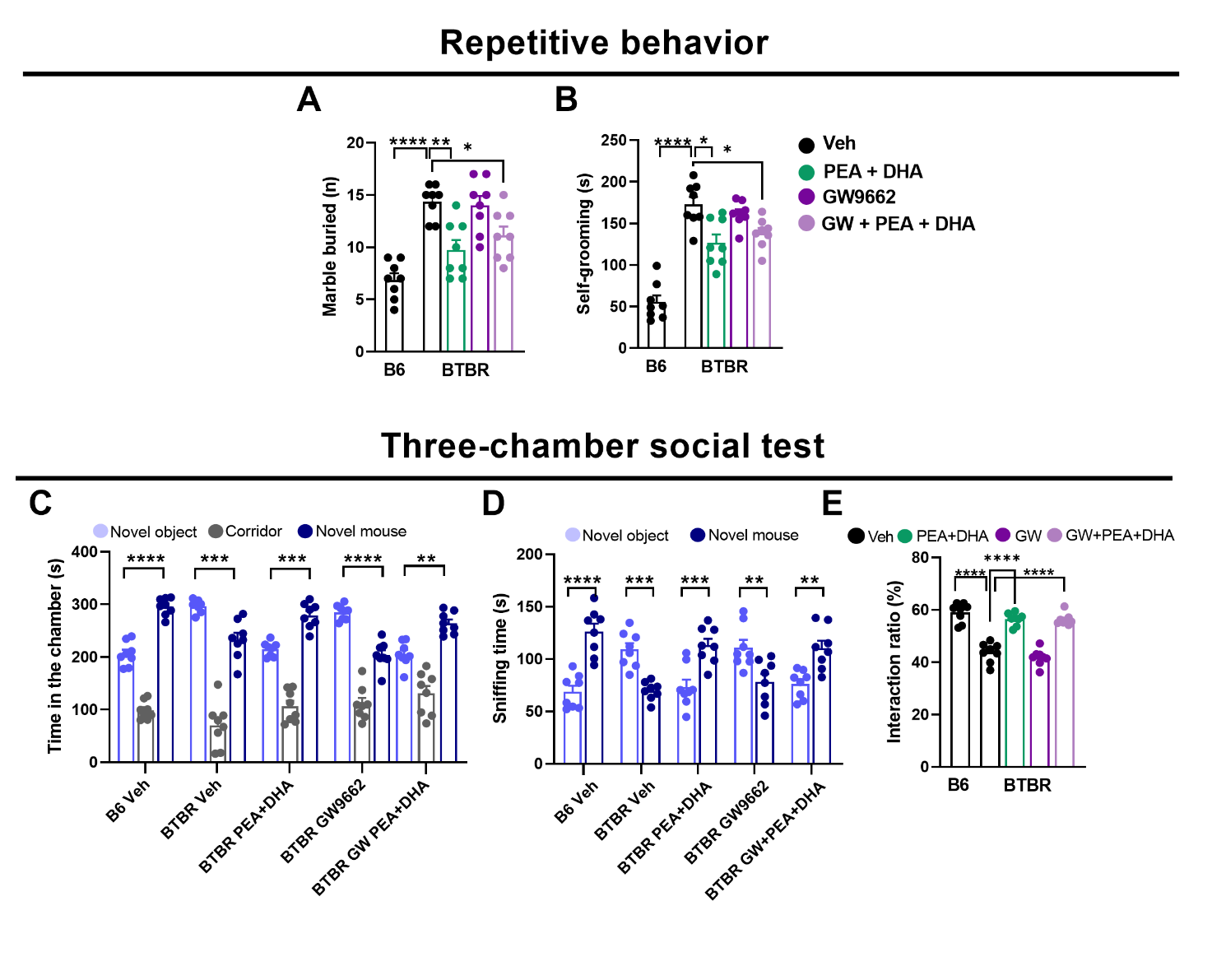


**Fig S1.** Neurosteroid levels in plasma and brain areas after PEA-um, DHA and PEA-um+ DHA co-treatment. (A) plasmatic, (B) cortical, and (C) hippocampal level of allopregnanolone in B6 and BTBR mice. (D) plasmatic, (E) cortical, and (F) hippocampal level of pregnenolone in B6 and BTBR mice treated with PEA-um+DHA. Data are presented as means ± S.E.M of n=5-8 mice/group. Statistical analysis was conducted by two-way ANOVA followed by Bonferroni’s post hoc-test. ****p*<*0.001, ***p*<*0.001, **p*<*0.01 and *p*<*0.05.

**Fig. S2.** Effects of PEA-um, DHA and PEA-um+ DHA co-treatment on ASD-like behaviors. (A) The number of buried marbles in the marble burying test. (B) Time mice spent grooming during the self-grooming test. (C) The amount of time spent in the chambers, (D) sniffing time and (E) preference index of time spent sniffing in the three-chamber social test. Number of (F) following, (G) push and crawl, (H) nose-to-nose and (I) nose-to-anogenital in the reciprocal social interaction test. Data are presented as means ± S.E.M of n=8 mice/group. Statistical analysis was conducted by one-way ANOVA followed by Bonferroni’s post hoc-test (A-B, E, F-I) or paired *t*-test (C-D). ****p*<*0.0001, ***p*<*0.001, **p*<*0.01 and *p*<*0.05.

**Fig. S3.** Role of PPAR-γ in PEA-um+DHA activity. (A) The number of buried marbles in the marble burying test. (B) Time mice spent grooming during the self-grooming test. (C) The amount of time spent in the chambers, (D) sniffing time and (E) preference index of time spent sniffing in the three-chamber social test. Data are presented as means ± S.E.M of n=8 mice/group. Statistical analysis was conducted by one-way ANOVA followed by Bonferroni’s post hoc-test (A-B, E) or paired t-test (C-D). ****p<0.0001, ***p<0.001, **p<0.01 and *p<0.05.

**Table 1. The antibodies used in this study.**

| **Antibody** | **Source (Cat no., brand)** | **Application** | **Dilution ratio** |
| --- | --- | --- | --- |
| SRD5A1 | 16884525, Thermofisher | WB | 1:1000 |
| Akr1c4 | 16888704, Thermofisher | WB | 1:1000 |
| PPAR-alpha | sc-398394, Santa Cruz | WB | 1:1000 |
| BDNF | ab108319, Abcam | WB | 1:1000 |
| TRKB | 4603, Cell Signaling | WB | 1:1000 |
| β-Actin | A5441, Sigma-Aldrich | WB | 1:5000 |

**Table 2.** **The sequences of primers used in this study.**

| **Gene** | **Forward primer (5’-3’)** | **Reverse primer (5’-3’)** |
| --- | --- | --- |
| Srd5a1  AKR1C4  PPAR-α  Tnf- α  IL-1β  IL-6  ccl2  Nlrp3  Bdnf  Ntrk2 | GAGTTGGATGAGTTGCGCCTA  GTGTGGTACTAAACGATGGTCAC  AGAGCCCCATCTGTCCTCTC  CCCTCACACCACTCAGATCATCTTCT  TAGTCCCTACCCCAATTTCC  TAGTCCTTCCTACCCCAATTTCC  TTAAAAACCTGGATCGGAACCAA  ATTACCCGCCCGAGAAAGG  TCATACTTCGGTTGCATGAAGG  CTGGGGCTTATGCCTGCTG | GGACCACTGCGAGGAGTAG  CAAATAAGCGGAGTCAAAATGGC  ACTGGTCTGCAAAACCAAA  GCTACGACGTGGGCTACAG  TTGGTCCTTAGCCACTCCTTC  TTGGTCAGCCACTCCTTC  GCATTAGCTTCAGATTTACGGGT  TCGCAGCAAAGATCCACACAG  AGACCTCTCGAACCTGCCC  AGGCTCAGTACACCAAATCCTA |
